# Structural and Optical Properties of La_1−*x*_Sr*_x_*TiO_3+δ_

**DOI:** 10.3390/ma7074982

**Published:** 2014-06-25

**Authors:** Lihong Gao, Zhuang Ma, Song Wang, Fuchi Wang, Cai Yang

**Affiliations:** School of Materials Science and Engineering, Beijing Institute of Technology, Beijing 100081, China; E-Mails: gaolihong@bit.edu.cn (L.G.); tonisamsong@foxmail.com (S.W.); wangfuchi@bit.edu.cn (F.W.); cai1102@126.com (C.Y.)

**Keywords:** perovskite structure, La_1−*x*_Sr*_x_*TiO_3+δ_, structural property, reflectivity

## Abstract

La_1−*x*_Sr*_x_*TiO_3+δ_ has attracted much attention as an important perovskite oxide. However, there are rare reports on its optical properties, especially reflectivity. In this paper, its structural and optical properties were studied. The X-ray diffraction, X-ray photoelectron spectroscopy, scanning electron microscopy and spectrophotometer were used to characterize the sample. The results show that with increasing Sr concentration, the number of TiO_6_ octahedral layers in each “slab” increases and the crystal structure changes from layered to cubic structure. A proper Sr doping (*x* = 0.1) can increase the reflectivity, reaching 95% in the near infrared range, which is comparable with metal Al measured in the same condition. This indicates its potential applications as optical protective coatings or anti-radiation materials at high temperatures.

## 1. Introduction

Perovskite structure oxides (ABO_3_) are of exceptional technological importance, due to their excellent physical properties, such as superconductivity, magnetism and ferroelectricity [1]. These properties could be significantly changed by distorting their crystal structure, doping the A or B site elements, introducing some defects or undergoing special conditions (high temperature, oxidizing or reduced atmosphere), *etc.* As an important perovskite structure oxide, A-site doped La_1−*x*_Sr*_x_*TiO_3+δ_ exhibits excellent chemical and physical stabilities. It has a wide variety of applications, such as electrodes, most often exploiting its good electrical conductive property. Its electrical property has been investigated by a number of studies [2,3,4,5]. As a 3d transition-metal oxide, La_1−*x*_Sr*_x_*TiO_3+δ_ shows metallic conductivity if properly doped [6,7,8]. Tokura *et al.* found a metal-insulator transition around *x* = 0.05 and the highest conductivity when *x* = 0.5 [6]. Besides, its crystal structure [9,10,11] and magnetic property [12] have also been intensively reported.

However, limited work has been done on the optical properties of La_1−*x*_Sr*_x_*TiO_3+δ_. Unfortunately, most of these investigations show its good transparency used as a transparent conducting oxide [2,9,13], and few studies were focused on improving its reflectance. For example, Yun’s [13] theoretical simulations suggested that La_0.125_Sr_0.875_TiO_3_ and La_0.125_Sr_1.875_TiO_4_ bulk materials have high transmittance (>80% above 400 nm in the visible range). T. Suzuki’ [14] and Zhu’s [2] experimental studies showed that the La_0.5_Sr_0.5_TiO_3+δ_ and La_0.4_Sr_0.6_TiO_3_ thin films are transparent (~80% transmittance in the visible range).

As required by some optical applications, such as protective coatings resisting high energy, laser equipment and protection components, the materials should have high reflectance and good chemical stability at high temperatures. As we all know, optical measurements can bring important information of materials such as the band structure, the density of states, and effective mass of the electron, which are related to the electrical properties of materials. Cho *et al.* [15] has investigated that the decrease of optical transmittance corresponds to an increase in the electrical conductivity in La_0.5_Sr_0.5_TiO_3_ thin film. This suggests that the optical properties are strongly related to the electrical properties. Besides, as suggested by our previous theoretical simulations [16], the reflectance of La_1−x_Sr_x_TiO_3+δ_ could reach 99% at 10.6 μm, if properly doped. This makes La_1−*x*_Sr*_x_*TiO_3+δ_ become a novel promising ceramic having high reflectivity. So, La_1−*x*_Sr*_x_*TiO_3+δ_, with a high reflectivity that is comparable with metals, is desirable.

In this paper, the structural and optical properties of La_1−*x*_Sr*_x_*TiO_3+δ_ in the visible/near infrared range are studied through experimental analysis. The effects of phase, energy band structure and density on its optical property are also discussed.

## 2. Experiments

La_1−*x*_Sr*_x_*TiO_3+δ_ samples were prepared in air by a conventional solid state reactions technique, with initial materials La_2_O_3_ (99.9% purity, Rare-chem hi-tech Co., Huizhou, China), SrCO_3_ (99.7% purity, Aladdin Industrial Corporation, Shanghai, China), and TiO_2_ (99.7% purity, Aladdin Industrial Corporation, Shanghai, China). Before weighing, La_2_O_3_ was pre-heated at 1000 °C for 2 h, and SrCO_3_, TiO_2_ were dried at 120 °C for at least 24 h. Powders were weighed by stoichiometric quantities and mixed in ethanol medium, and subsequently ball-milled in a planetary mill for 6–8 h. After drying and sieving, the mixtures were pre-sintered at 1200–1300 °C for 2 h. The powders were pre-pressed into tablets at 8–10 MPa and then pressed at 200 MPa by cold isostatic press. Afterwards, the tablets were sintered at 1550 °C for 10 h with a heating rate of 5 °C/min. In order to prepare the La_0.9_Sr_0.1_TiO_3+δ_ samples with different densities, the tablets were sintered at 1550 °C for 5 h, 10 h, 1600 °C for 5 h, 10 h, respectively.

The density of sintered samples was tested by Mettler-Toledo AL/AB-N analytical balance. The phase structures were characterized by X-ray diffraction (XRD, X’Pert PRO MPD, PANalytical, Almelo, The Netherlands) with Cu Kα radiation and analyzed by JADE (version 5.0). The valence band spectra were analyzed by X-ray photoelectron spectroscopy (XPS, PHI 5300 ESCA, Perkin Elmer, CA, USA). The surface morphology was characterized by scanning electron microscopy (SEM, S-4800, Hitachi, Tokyo, Japan). The reflectance was measured by a commercial spectrophotometer (Cary 5000, Varian, CA, USA) operating in the UV/VIS/NIR spectral range using a 110 mm integrating sphere.

## 3. Results and Discussion

### 3.1. Phase and Microstructure Characterization

**Figure 1:**
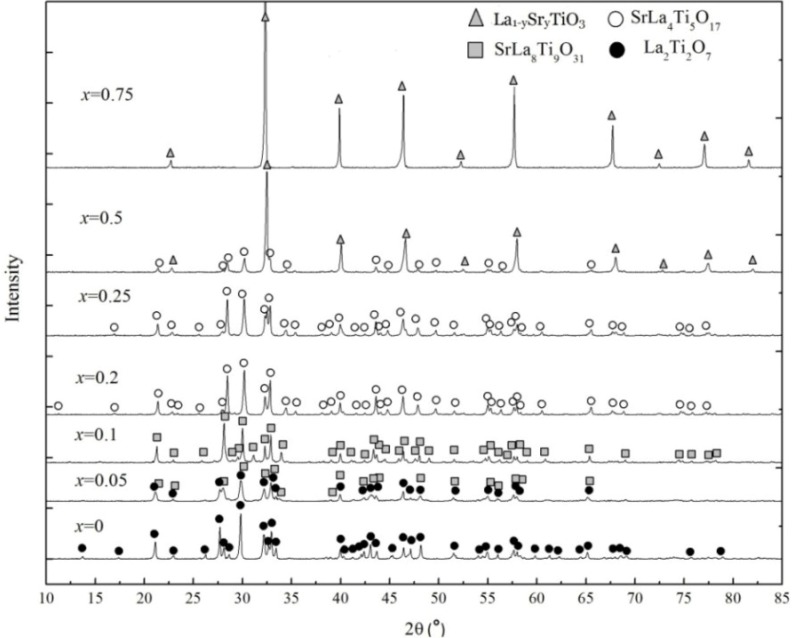
XRD patterns of La_1−*x*_Sr*_x_*TiO_3+δ_ as functions of *x* (0 ≤ *x* <1).

The phase of La_1−*x*_Sr*_x_*TiO_3+δ_ changes with varying Sr concentration, as suggested by the XRD patterns in Figure 1. When *x* ≤ 0.1, the phase transfers La_2_Ti_2_O_7_ to SrLa_8_Ti_9_O_31_. La_2_Ti_2_O_7_ is produced by the chemical reaction of initial materials,

La_2_O_3_ + 2TiO_2_ → La_2_Ti_2_O_7_ (1)

Because this sample is prepared in air (oxide environment) and Ti^+4^ is more stable than Ti^+3^, the electro neutrality will be preferably maintained by the ionic compensation [11], *i.e.*, the introduction of O^−2^, which makes δ equal to 0.5. Therefore, LaTiO_3.5_ (La_2_Ti_2_O_7_) is produced rather than LaTiO_3_. When 0.2 ≤ *x* ≤ 0.25, the XRD pattern strongly resembles that of La_5_Ti_5_O_17_, which is surely impossible in this case. This suggests that the two, *i.e.*, the phase in this study and La_5_Ti_5_O_17_, may be structurally analogous. According to M.E. Bowden’s studies [17], it is SrLa_4_Ti_5_O_17_ that belongs to the same family of A*_m_*B*_m_*O_3*m*+2_ with La_5_Ti_5_O_17_. When *x* = 0.5, two phases exist, SrLa_4_Ti_5_O_17_ and La_1−*y*_Sr*_y_*TiO_3+δ_(0.5 < *y* < 1). This fact indicates that even though Sr and La have the biggest solubility among rare earth elements dopants for the A site of La_1−*x*_Sr*_x_*TiO_3+δ_, it is impossible to dope illimitably. If Sr concentration exceeds the maximum solubility, SrTiO_3_ is easily produced by the reaction of initial materials.

SrCO_3_ + TiO_2_ → SrTiO_3_+CO_2_↑ (2)

La partially substitutes Sr producing La_1−*y*_Sr*_y_*TiO_3+δ_. When *x* = 0.75, La_0.25_Sr_0.75_TiO_3_ is obtained.

The obtained lattice parameters from JADE are listed in Table 1. It can be seen that they are in rather good agreement with other reports.

**Table 1:** Lattice parameters of La_1−*x*_Sr*_x_*TiO_3+δ_ as functions of *x* (0 ≤ *x* < 1).

*x*	Phase	Lattice Parameters	Reported Values
0	La_2_Ti_2_O_7_	*a* = 7.817 Å, *b* = 13.051 Å, *c* = 5.546 Å, α = 90°, β = 98.64°, γ = 90°	*a* = 7.800 Å, *b* = 13.01 Å, *c* = 5.55 Å, α = 90°, β = 98.6°, γ = 90° [11]
0.1	SrLa_8_Ti_9_O_31_	*a* = 7.779 Å, *b* = 56.853 Å, *c* = 5.536 Å, α = 90°, β = 90°, γ = 90°	*a* = 7.81 Å, *b* = 57.01 Å, *c* = 5.533 Å, α = 90°, β = 90°, γ = 90° [17]
0.25	SrLa_4_Ti_5_O_17_	*a* = 3.918 Å, *b* = 5.531 Å, *c* = 31.514 Å, α = 90°, β = 97.12°, γ = 90°	*a* = 3.982 Å, *b* = 5.532 Å, *c* = 31.466 Å, α = 90°, β = 97.14°, γ = 90° [18]
0.75	La_0.25_Sr_0.75_TiO_3_	*a* = *b* = *c* = 3.911 Å, α = β = γ = 90°	*a* = *b* = *c* = 3.905 Å, α = β = γ = 90° [19]

**Figure 2:**
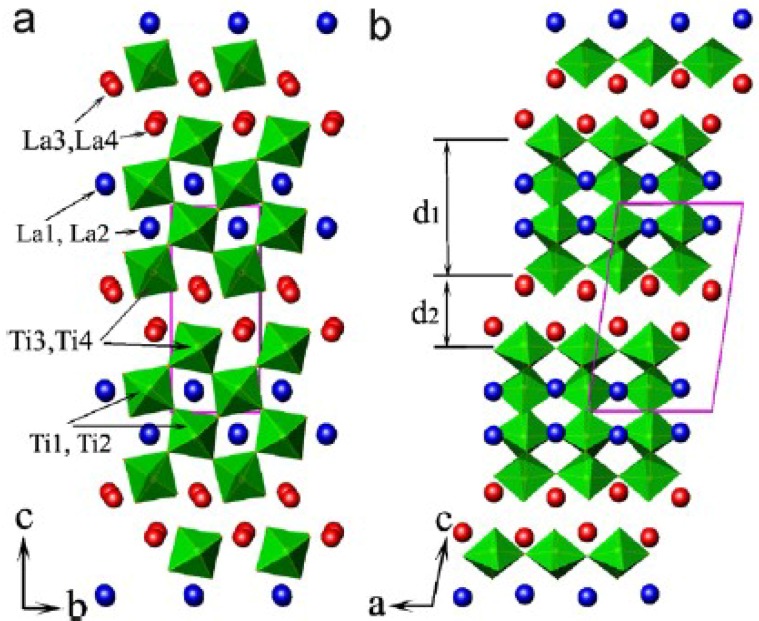
Schematic crystal structure of La_2_Ti_2_O_7_ projected along (**a**) the [100] and (**b**) [010] directions, reprinted with permission from [20]. Copyright 2006 Elsevier.

The ideal perovskite structure is cubic, but it will be distorted or changed by some defects, or substitution, *etc.* La_2_Ti_2_O_7_, SrLa_8_Ti_9_O_31_ and SrLa_4_Ti_5_O_17_ are monoclinic and orthorhombic, as shown in Table 2. They belong to layered perovskite structure A*_m_*B*_m_*O_3*m*+2_ with *m* = 4, 4.5 and 5, respectively (*m* is the number of TiO_6_ octahedral layers in each “slab”). Figure 2 shows the schematic crystal structure of La_2_Ti_2_O_7_ projected along the [100] and [010] directions. The perovskite “slab” contains four layers of TiO_6_ octahedra. Adjacent slabs are offset from one another by half of one TiO_6_ octahedron height, and the octahedron connectivity is broken at the shear interface. So, the octahedra in different slabs are separated by two layers of La cations, La3 and La4 [20,21]. The thickness of slabs (d1, d2) are determined by the number of layers.

Similarly, for SrLa_4_Ti_5_O_17_, each slab contains 5 layers of TiO_6_ octahedra, but part of La is substituted by Sr. SrLa_8_Ti_9_O_31_ is the transitional phase between La_2_Ti_2_O_7_ and SrLa_4_Ti_5_O_17_, constituted by the alternate slabs of La_2_Ti_2_O_7_ and SrLa_4_Ti_5_O_17_. So *m* is equal to 4.5 [17].

**Table 2:** Phases and crystal structures of La_1−*x*_Sr*_x_*TiO_3+δ_ as functions of *x* (0 ≤ *x* <1).

*x*	Phase	Crystal Structure	Crystal System
0	La_2_Ti_2_O_7_	Layered structure *m* = 4	Monoclinic
0.05	La_2_Ti_2_O_7_	Layered structure *m* = 4	Monoclinic
	SrLa_8_Ti_9_O_31_	Layered structure *m* = 4.5	Orthorhombic
0.1	SrLa_8_Ti_9_O_31_	Layered structure *m* = 4.5	Orthorhombic
0.2	SrLa_4_Ti_5_O_17_	Layered structure *m* = 5	Monoclinic
0.25	SrLa_4_Ti_5_O_17_	Layered structure *m* = 5	Monoclinic
0.5	SrLa_4_Ti_5_O_17_	Layered structure *m* = 5	Monoclinic
	La_1−y_Sr_y_TiO_3_ (0.5 < *y* < 1)	–	Cubic
0.75	La_0.25_Sr_0.75_TiO_3_	–	Cubic

La_1−*y*_Sr*_y_*TiO_3+δ_ and La_0.25_Sr_0.75_TiO_3_ are cubic structure, *i.e.*, *m* = ∞. The La substitution for Sr either does not change or seldom changes the structure of SrTiO_3_, maintaining its cubic form.

Consequently, with increasing the Sr concentration, *m* increases and the crystal structure changes from layered to cubic structure with a critical concentration of approximately 0.5.

**Figure 3:**
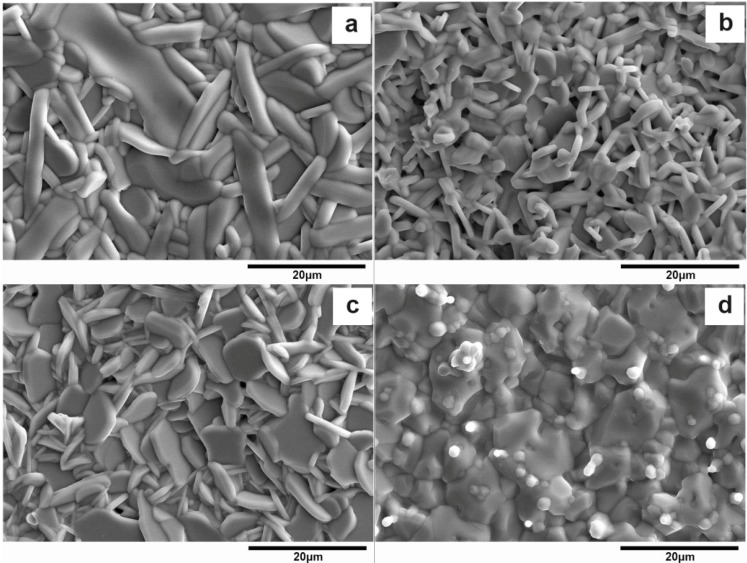
SEM morphology of La_1−*x*_Sr*_x_*TiO_3+δ_ **(a**) *x* = 0; (**b**) *x* = 0.1; (**c**) *x* = 0.25; (**d**) *x* = 0.75.

The morphology of La_1−*x*_Sr*_x_*TiO_3+δ_ is shown in Figure 3. It can be seen that the crystal grain shows an asymmetric grain structure for 0 ≤ *x* ≤ 0.25 and equiaxial structure for *x* = 0.75. This fact is in good agreement with their crystal phase composition. In terms of their grain size, in Figure 3a the average thickness of grains is about 2–4 μm and the aspect ratio is about 5–8, followed by Figure 3c (the thickness is about 1–2 μm and the aspect ratio is about 4–6). Figure 3b has the smallest grain size (the thickness is about 0.5–1 μm and the aspect ratio is about 3–5). Finally, the average diameter in Figure 3d is about 10 μm.

### 3.2. Reflectivity and Its Effect Factors

#### 3.2.1. Reflectivity

Using the same sample, we measured its roughness and reflectivity before and after polishing. The roughness, which is the average value of 10 measurements at different spots, is Ra0.5 μm and Ra0.1 μm before and after polishing, respectively. However, their reflectivity difference is smaller than 1.5%. So the roughness has very small and negligible effect on the reflectivity for this material. In order to simplify the procedure of using this material as high reflective material or coatings, we did not further polish the samples and kept a raw surface to study its reflectivity property.

**Figure 4:**
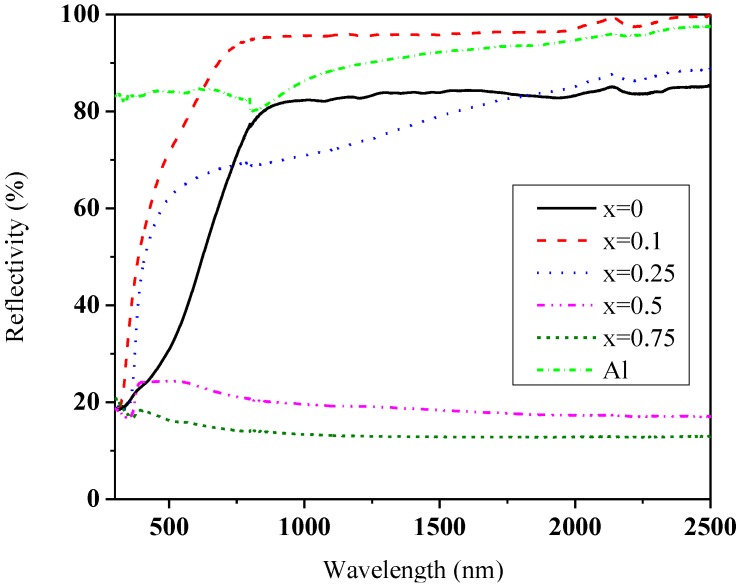
Reflectivity of La_1−*x*_Sr*_x_*TiO_3+δ_.

The reflectivity of La_1−*x*_Sr*_x_*TiO_3+δ_, without any surface treatments, is shown in Figure 4. It can be seen that the reflectivity of La_1−*x*_Sr*_x_*TiO_3+δ_ increases in the visible range. The reflectivity of un-doped La_2_Ti_2_O_7_ is about 75%–85% in the neat infrared range of 780–2500 nm. After doping Sr, a low Sr concentration (*x* = 0.1) makes the reflectivity increase obviously, above 95% in the near infrared range (>815 nm), which is rather rare among bulk ceramics. It can be seen that its reflectivity is very steady as functions of wavelength and even higher than that of metal Al, which was measured in the same condition. A higher Sr concentration (*x* = 0.25) improves the reflectivity only at the long wavelengths (>1820 nm), and the reflective values increase as functions of wavelength (<89%). A high Sr concentration, *x* > 0.5, is unfavorable to the improvement of reflectivity (<25%). Consequently, a proper dopant of Sr can increase the reflectivity of La_1−*x*_Sr*_x_*TiO_3+δ_, reaching 95% comparable with Al, but one should take care of its concentration.

Considering the reflectivity value was measured in an integrating sphere, thus we have measured total hemispherical reflectance including specular, diffuse reflectance and scatter. Even though some materials with a higher total reflectance than Al, such as pressed Polytetrafluoroethylene polymer (Spectralon), exist, they have poor stability. The Polytetrafluoroethylene polymer has a melting point of 327 °C which is rather lower than that of most of ceramics. So La_1−*x*_Sr*_x_*TiO_3+δ_ with a high reflectivity has potential applications at high temperatures.

#### 3.2.2. Effect of Phase Structure on the Reflectivity of La_1−*x*_Sr*_x_*TiO_3+δ_

Several factors could have effects on the reflectivity of La_1−*x*_Sr*_x_*TiO_3+δ_, such as its micro-factors (microstructure, element composition, phase structure, energy band structure, *etc.*) and macro-factors (surface, density, *etc.*). However, in this study, we focus on the effects of phase structure, energy band structure and density.

**Figure 5:**
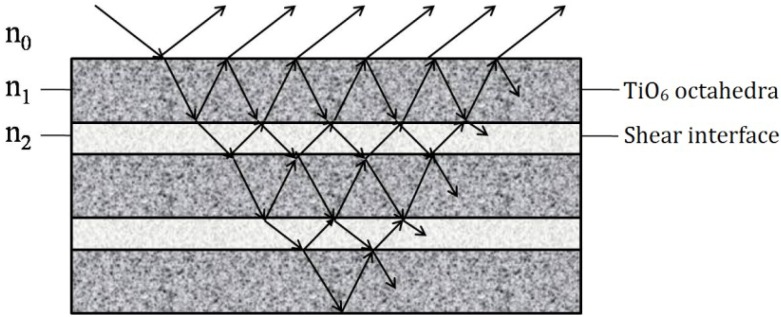
Schematic reflectance in the layered perovskite structure.

Comparing Table 2 and Figure 4, it could be concluded that the layered perovskite structure is more beneficial to the reflectivity than cubic structure, and SrLa_8_Ti_9_O_31_ enhances a higher value of reflectivity than La_2_Ti_2_O_7_ and SrLa_4_Ti_5_O_17_. This may be because the layered structure could be regarded to be composed of layers with refractive index *n*_1_ and *n*_2_, as shown in Figure 5. The reflectivity (*R*) at the interface of *n*_1_/*n*_2_, *i.e.*, the light propagates from *n*_1_ to *n*_2_, can be expressed as follows:

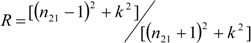
(3)
where *n*_2__1_ = *n*_2_/*n*_1_. For the dielectric materials, *k* ≈ 0 at the visible/near infrared wavelengths, Equation (3) will then induce to:

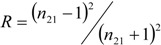
(4)


Therefore, the refractive index difference of layers leads to more reflection process and the reflectivity can be thus improved by multi-reflectance at the interface, which is like the optical multilayer thin films.

**Figure 6:**
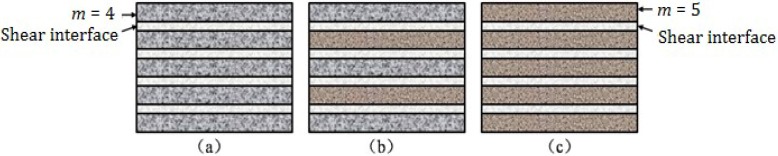
Skematic structures of La_2_Ti_2_O_7_, SrLa_8_Ti_9_O_31_ and SrLa_4_Ti_5_O_17_ (**a**) La_2_Ti_2_O_7_; (**b**) SrLa_8_Ti_9_O_31_; (**c**) SrLa_4_Ti_5_O_17_.

Figure 6 shows the schematic structure of La_2_Ti_2_O_7_, SrLa_8_Ti_9_O_31_ and SrLa_4_Ti_5_O_17_. SrLa_8_Ti_9_O_31_ consists of alternate La_2_Ti_2_O_7_ (*m* = 4) and SrLa_4_Ti_5_O_17_ (*m* = 5), so this “multilayer thin film” is composed of three layers with different thicknesses. Its interface is more complicated which may be favorable to a higher reflectivity. The periodicity of the alternating layers are much smaller than the wavelength of light, thus the contribution of the proposed multiple interfaces will be limited.

#### 3.2.3. Effect of Energy Band Structure on the Reflectivity of La_1−*x*_Sr*_x_*TiO_3+δ_

**Figure 7:**
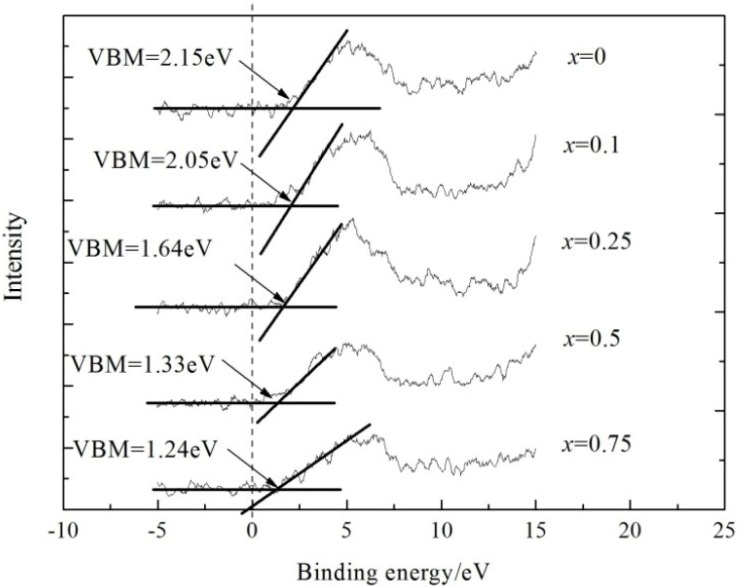
Valence band XPS spectra of La_1−*x*_Sr*_x_*TiO_3+δ_.

The valence band plays a key role in the energy band structure and optical property during the electronic inter-band transition. Based on the XPS measured binding energy of the core levels, it is possible to align the valence band XPS spectrum to a common energy scale with respect to the Fermi level. The valence band XPS spectrum could fix the energy at the top of valence band, *i.e.*, valence band maximum (VBM) [22]. The VBM of La_1−*x*_Sr*_x_*TiO_3+δ_ is shown in Figure 7, where the Fermi level is located in the energy of 0 eV. It can be seen that VBM moves to the Fermi level with increasing the Sr concentration. This means the top energy of electrons becomes bigger with increasing Sr concentration. This suggests that the Sr doping makes the electrons in the valence band of La_1−*x*_Sr*_x_*TiO_3+δ_ require less energy to be excited, *i.e.*, jumping from the valence band to a higher level. The reflection occurs when the excited electrons in the higher energy band fall down to the lower energy band. So, the less energy that is required is beneficial to the inter-band transitions and reflectivity. However, the energy band structure could not yet decide their final optical property, due to several factors.

#### 3.2.4. Effect of Density on the Reflectivity of La_1−*x*_Sr*_x_*TiO_3+δ_

**Figure 8:**
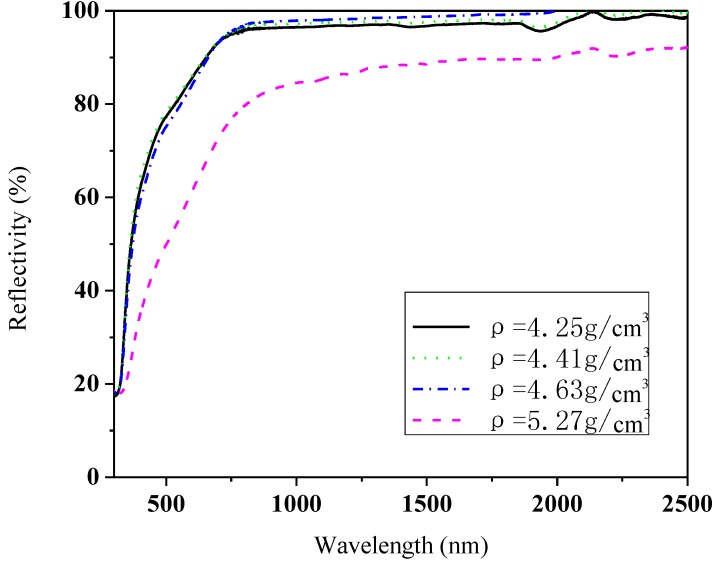
Reflectivity of La_0.9_Sr_0.1_TiO_3+δ_ with different densities.

Even for the same phase composition and energy band structure as La_0.9_Sr_0.1_TiO_3+δ_, its different densities could lead to different reflectivity. The different sintering conditions of 1550 °C for 5 h, 10 h, 1600 °C for 5, 10 h, correspond to the density of 4.25 g/cm^3^, 4.41 g/cm^3^, 4.63 g/cm^3^, and 5.27 g/cm^3^ respectively. A high temperature and a long time favor a dense material. As shown in Figure 8, the sample with density 4.63 g/cm^3^ has the highest reflectivity (>95% in the near infrared range above 815 nm). The one with the highest density 5.27 g/cm^3^ has the lowest reflectivity (80%–92%), about 10% lower than that of 4.63 g/cm^3^. Therefore, the densest possible materials are not always beneficial to improve the reflectivity. On the other hand, a too low density could also decrease its reflectivity (e.g., 4.25 g/cm^3^).

**Figure 9:**
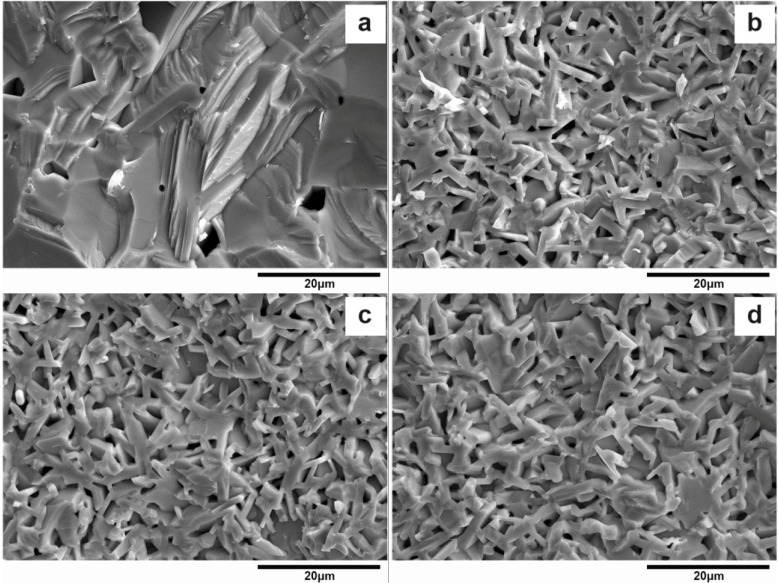
SEM cross-section morphology of La_0.9_Sr_0.1_TiO_3+δ_ with different densities (**a**) 5.27 g/cm^3^; (**b**) 4.63 g/cm^3^; (**c**) 4.41 g/cm^3^; (**d**) 4.25 g/cm^3^.

It can be seen from the cross-section morphology in Figure 9 that La_0.9_Sr_0.1_TiO_3+δ_ in Figure 9a is obviously denser than others, as also suggested by their values of densities. Its grains have grown much bigger and the grain boundaries are almost invisible. However, the grains in Figure 9b–d are smaller (2–3 μm in thickness) and the pores are also small and uniform. Their reflectivity values in Figure 8 suggest that the uniformity and small size of grains and pores are favorable to the reflectivity. This is because the pores, as a secondary phase with refractive index about 1, could cause the refractive index difference in the material. Thus, a proper quantity and shape of pores can increase the reflectivity.

## 4. Conclusions

This paper has investigated the structural and optical property of La_1−*x*_Sr*_x_*TiO_3+δ_ (0 ≤ *x* < 1). Results show that when *x* < 0.5, La_1−*x*_Sr*_x_*TiO_3+δ_ belongs to the same family of A*_m_*B*_m_*O_3*m*+2_ and *m* increases with increasing the Sr concentration. When *x* > 0.5, the crystal structure changes from layered to cubic structure, as suggested by the morphology. A proper dopant of Sr (*x* = 0.1) can increase the reflectivity of La_1−*x*_Sr*_x_*TiO_3+δ_, reaching 95% in the near infrared range which is comparable with metal Al. This high reflectivity is attributed to its layered perovskite structure, movement of the top of valence band to the Fermi level, proper density, uniformity and small size of grains and pores. Its high reflectivity indicates its potential applications as optical protective coating or anti-radiation materials at high temperatures.
